# A Gene-Expression Based Comparison of Murine and Human Inhibitory Interneurons in the Cerebellar Cortex and Nuclei

**DOI:** 10.1007/s12311-025-01809-y

**Published:** 2025-02-28

**Authors:** Karl Schilling

**Affiliations:** Anatomisches Institut– Anatomie und Zellbiologie, Rheinische Friedrich-Wilhelms-Universität Bonn, Nussallee 10, D53115 Bonn, Germany

**Keywords:** Cerebellar cortex, Cerebellar nuclei, Inhibitory interneuron, Human, Mouse, Gene expression analysis

## Abstract

Cerebellar information processing is critically shaped by several types of inhibitory interneurons forming various intra-cerebellar feed-forward and feed-back loops. Evidence gathered over the past decades has focused interest on a non-uniform set of cortical inhibitory interneurons distinct from “classical” Golgi, basket or stellate cells, summarily referred to as PLIs (for Purkinje cell layer interneurons). Similarly, cerebellar nuclear inhibitory interneurons have gained increasing attention. Our understanding of the functions of these cells is still fragmentary. For humans, we lack functional data, and even any dependable morphological classification for these cells. Here, I used publicly available single cell based gene expression data to compare inhibitory interneurons from the cerebellar cortex and inhibitory nuclear neurons of humans and mice. Integration of nuclear and cortical cells revealed transcriptomic similarities between subsets of these cells and suggest known characteristics of cortical cell types may be helpful to devise strategies for the further characterization of nuclear inhibitory interneurons. Comparison of human and murine PLIs indicate that these strongly differ by the expression of genes used to characterize these cells in mice. This limits their utility to identify and classify human PLIs, and leaves the question open as to the number and characteristics of non-Golgi inhibitory interneurons resident in the cerebellar granule cell and Purkinje cell layers in humans.

**Supplementary Information:**

The online version contains supplementary material available at 10.1007/s12311-025-01809-y.

## Introduction

Since the fundamental theories by Albus [1] and Marr [2] underpinning much cerebellar research in the past decades have been published, our views of cerebellar structure, function, and pathology have rapidly evolved. Traditionally seen as specific for motor control and learning, we now appreciate, and try to understand, the cerebellum’s role in perceptual processing, affect and social cognition, and in clinical conditions which comprise, beyond ataxias, also neuropsychiatric affections [3–7].

This evolving perception of cerebellar functions builds largely on experimental studies performed in animal models, notably rodents. These studies led to a detailed understanding of the extensive wiring of the cerebellum with functionally diverse parts of the extra-cerebellar CNS (for reviews, see [8–12]). They also elucidated the crucial role of cerebellar nuclei for intracerebellar processing and plasticity (for a recent review, see [9]) and, last but not least, revealed that cerebellar cell types defined by classical histology are far more diverse than traditionally thought once their molecular diversity is accounted for [13–15].

The differential expression of the zebrin II antigen [16, 17] in spatially segregated subsets of Purkinje cells offered early clues into how the molecular diversity of a classical cell type might relate to cerebellar functions (see also [18–20] for further examples and discussion). Similarly, granule neurons, once viewed as forming a highly homogeneous set of cells, are now known to comprise molecularly distinct subsets organized in defined modules (for an enlightening discussion of the potential developmental origin of granule cell compartments, see [21]). Further, converging morphological [22, 23, 29], molecular [24, 25] and functional data [26, 27] indicate that cerebellar cortical inhibitory interneurons also are far more diverse than suggested by their traditional classification as Golgi, basket and stellate cells (reviewed in [28]). Lastly, the recent molecular characterization of cerebellar nuclear cells also opened up a novel avenue towards a better understanding of the functional integration of cerebellar nuclei and cerebellar cortex [9, 30].

Whereas the rodent cerebellum allows a multifaceted experimental approach, access to the human cerebellum and analysis of its cellular diversity, in particular that of inhibitory cerebellar neurons, is more limited. Still, the molecular description of individual human cerebellar cells recently achieved [25, 30, 31] generated data that should allow approaching at least some of the open questions.

Here, we combined and integrated single cell RNA (scRNA) data for human and murine cerebellar cortical inhibitory interneurons and nuclear inhibitory neurons [25, 30, 31] to compare and contrast these cells. Our analysis points at gene expression-defined similarities between nuclear inhibitory neurons and distinct sets of morphologically and functionally characterized sets of cortical inhibitory interneurons. Comparisons of human and murine data also highlight human specific features, as well as deficits in our understanding of human cell types.

## Materials and Methods

### Data Processing

Data processing was done using R (version 4.4.1; R Core Team, 2024; available at https://cran.r-project.org/) and package Seurat (version 5.10). Further packages used included sctransform, glmGamPoi, patchwork, reticulate, rliger, scCustomize, tidyverse, viridis (all available from https://cran.r-project.org/), EnsDb.Hsapiens.v86 (available at https://www.bioconductor.org/), Seurat.utils (https://github.com/vertesy/Seurat.utils), and RightOmicsTools (https://github.com/Alexis-Varin/RightOmicsTools). For the calculation of cross-entropy values, we followed the procedure outlined by Roca et al. [76] building on the code provided with this publication.

### Data Sources and Preliminary Processing

Expression data for adult human cerebellar nuclear cells published by Kebschull et al. [30] were obtained from the NCBI Gene Expression Omnibus (GEO; accession numbers GSE160471, samples GSM4873766, file “GSM4873766_human_data.RData”). Data for inhibitory cells were isolated as per the annotation (“inh”) of the authors.

Expression data for the adult human cerebellar cortex [25] were downloaded from the GEO repository (GSE165371; file “GSE165371_human_combined.tar.gz”). Annotations for these data as used in the original publication were obtained from the Broad Institute Single Cell Portal (https://singlecell.broadinstitute.org/single_cell; files “mouse_human_joint_golgi_obj.RDS” and “mouse_human_joint_mli_pli_obj.RDS”). Data for inhibitory cells were isolated as per the authors’ annotation (“mli-pli” and “golgi”). These data comprised a complement of cells characterized by distinctly low numbers (< 1500) of expressed genes (features), and a high fraction of expressed mitochondrial genes (> 10%) that consistently clustered well separate from cells expressing higher numbers of genes (features). These cells (clusters) were excluded from further analyses.

Gene expression data for the adult human cerebellar cortex and nuclei published by Siletti et al. [31] were obtained from the cellxgene website (https://cellxgene.cziscience.com/). They included those referred to as “Dissection: Cerebellum (CB) - Cerebellar Vermis - CBV”, https://datasets.cellxgene.cziscience.com/29e0b90a-fd4e-4546-99fe-4c4f6a09d511.rds; those referred to as “Dissection: Cerebellum (CB) - Lateral hemisphere of cerebellum - CBL”, https://datasets.cellxgene.cziscience.com/2173c001-3778-4d46-8383-5e6903095f05.rds; and those referred to as Dissection: Cerebellum (CB) - Cerebellar deep nuclei– CbDN, https://datasets.cellxgene.cziscience.com/feb8bd98-bc8a-4e4e-83e3-a820f65aa3d2.rds.

From these sets, we isolated cells annotated by the supercluster term “Cerebellar Inhibitory”. Additional cerebellar inhibitory neurons forming part of the supercluster “Splatter” (a heterogenous cluster comprising multiple cell types and named simply for its form in the original analysis; see [31]) were isolated based on the expression of established marker genes for GABAergic and/or glycinergic neurons (GAD2 and/or SLC32A1 and/or SLC6A5). We also verified that these cells did not express typical markers of excitatory neurons. Eventually, cells originating from cerebellar dissections and annotated as members of clusters 298–307, 362, 365 and 380 were included for further analyses. Of these, clusters 298–307 were part of the supercluster “Cerebellar Inhibitory”, and clusters 362, 365 and 380 were part of the supercluster “Splatter”.

Gene annotations of the data from Kebschull et al. [30] and Kozareva et al. [25], which were given as HGNC symbols, were converted to ENSEMBL IDs using the R package EnsDb.Hsapiens.v86 to allow their integration with the data of Siletti et al. [31]. As a final step prior to integration, the individual data sets were subset such that they all shared the same set of genes, and genes expressed in fewer than three cells (in the combined data sets) were removed.

### Integration of Cortical and Nuclear Inhibitory Interneurons

For data integration, original data sets were first split as per the batches constituting each of these data sets. They were then combined in one Seurat (v5) object. Prior to integration, one batch from the data of Siletti et al. [31] (original identity # 10 × 176) was removed as it comprised too few cells (two) to allow sensible integration.

To account for different sequence depth of the data set analyzed, we used sctransform ([32]; see also https://github.com/hbctraining/scRNA-seq/blob/master/lessons/06_SC_SCT_and_integration.md) for normalization and variance stabilization of count data, followed by principal component analysis (PCA) as per the standard Seurat workflow. We initially compared various integration methods (canonical correlation analysis (CCA); reciprocal PCA (RPCA); joint PCA (JPCA); and Harmony [33] (all implemented in Seurat), and ultimately used Harmony for batch-effect correction, given its ability to integrate data assayed with different technologies and to merge common cell-types across batches while keeping distinct cell-types apart [33, 34] (see also [35] for a recent methodological comparison). Following integration, data were subjected to dimensionality reduction using both uniform manifold approximation and projection (umap) and t-distributed stochastic neighbor embedding (tSNE) as implemented in package Seurat. tSNE was run on 30 PCA dimensions using Seurat’s standard settings (perplexity = 30, learning rate = 200, maximum number of iterations = 1000, theta = 0.5). For figure generation, colors optimized for readers with impaired color vision were used, based on the Okabe-Ito palette of the R package grDevices, and the cividis palette of the R package viridis.

**Fig. 1:**
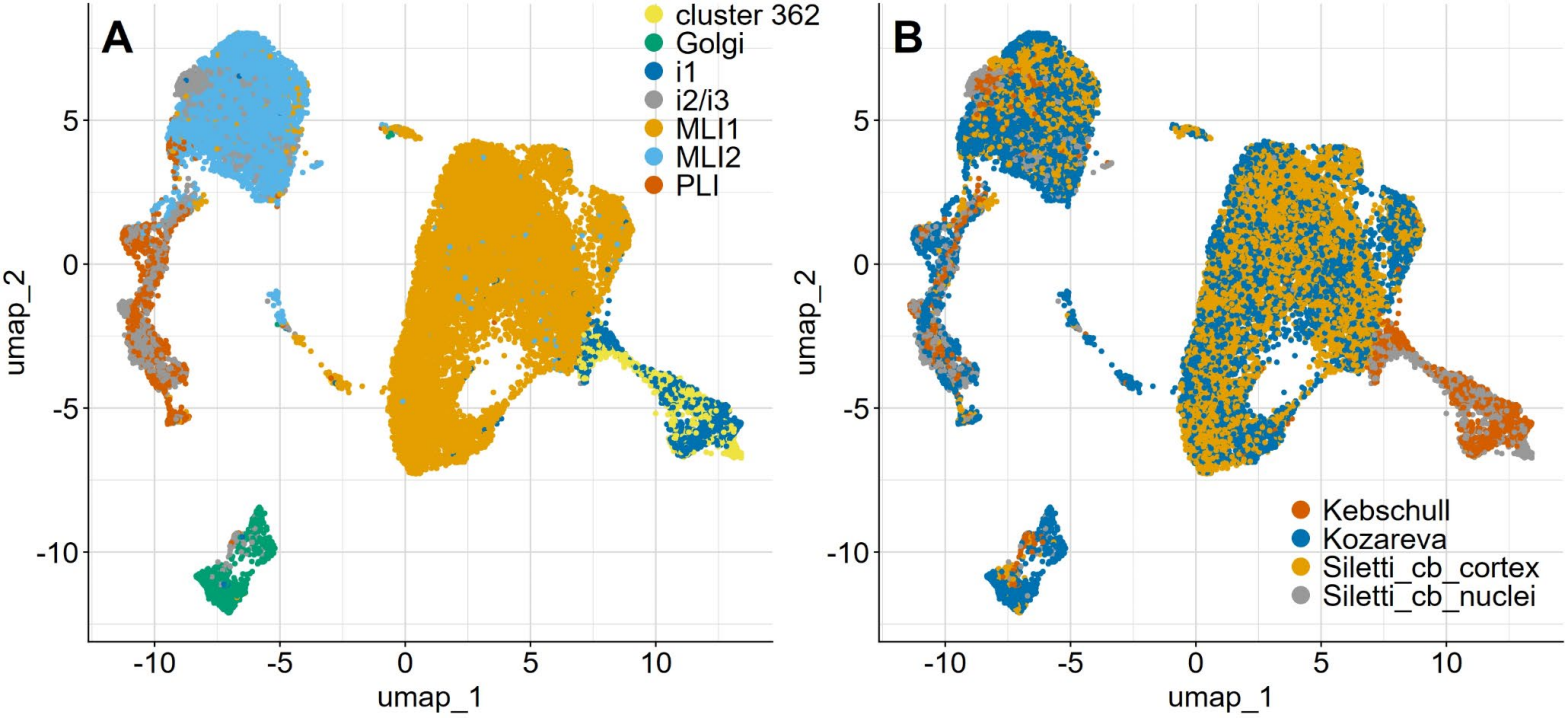
Integration of human cerebellar cortical and nuclear inhibitory neurons reveals cell type-specific clustering independent of data source. **A**) Following integration with nuclear inhibitory neurons, cortical molecular layer (MLI1, MLI2), neurons, Golgi cells and Purkinje cell layer interneurons form well separated clusters. Nuclear interneurons of classes i2 and i3 (i2/i3) overlap with Purkinje cell layer interneurons (PLI), molecular layer interneurons of type 2 (MLI2), and also with Golgi cells. Nuclear cells of type i1 and cluster 362 are clearly separated from other cerebellar inhibitory neurons. **B**) Efficient data integration is documented by the extensive cell type-specific overlap between data originating from the various sources used (Kozareva et al. [25]) for cortical, Kebschull et al. [30] for nuclear, and Siletti et al. [31] for cortical and nuclear cells). Note also that the overlap between nuclear and cortical cells is not dependent on the data sources

Filtering of the original data sets from Kebschull et al. [30], Kozareva et al. [25] and Siletti et al. [31] as detailed above resulted in a set of 25,840 cells and 19,121 common genes (features) expressed in all three data sets. Of these cells, 4,270 originated from dissections of the cerebellar nuclei and 21,570 from the cerebellar cortex. Further details about the origin and classification of these cells are given in the legend of Fig. 1 and Supplementary Table 1.

### Data for Murine Cells

Expression data for adult murine cerebellar nuclear cells published by Kebschull et al. [30]) were obtained from the NCBI Gene Expression Omnibus (GEO; accession numbers GSE160471, file “GSM4873765_mouse_data.RData.gz”). Expression data for adult murine cerebellar cortex [25]) were downloaded from the GEO repository (GSE165371; file “GSE165371_cb_adult_mouse.tar.gz”). Inhibitory neurons from these sets were isolated as per the annotations of the authors, and a random sample of 12,000 cortical cells was used for integration with nuclear cells. Integration and all subsequent analyses of murine cell followed the procedures outlined for human cells. Further details about the origin and classification of these cells are given in Supplementary Table 2.

Homologous genes in human and murine data sets were identified using the MGI Vertebrate Homology database (as of 2024-09-09; see https://www.informatics.jax.org/downloads/reports/HOM_MouseHumanSequence.rpt).

## Results

### General

Differential gene expression in the integrated data sets of human cortical and nuclear cerebellar neurons allowed the efficient separation of distinct types of cerebellar cortical inhibitory interneurons (Fig. 1A). Further, deep nuclear inhibitory neurons of class i1 as defined by Kebschull et al. [30] and those of cluster 362 of Siletti et al. [31] were well separated from all other cells, but extensively overlapped with each other. Figure 1A and B also document the efficacy of the integration strategy chosen, as molecular layer interneurons of type 1 and 2 (MLI1 and MLI2 for short), Golgi cells, and Purkinje cell layer inhibitory interneurons, (PLI) partitioned efficiently by cell type, irrespective of their origin from the Kozareva et al. [25] or Siletti et al. [31] data sets. Yet one caveat should be mentioned here: as closer inspection of Fig. 1A reveals, a few cells originally described as MLI2, and a very few described as PLI cells clustered actually with MLI1 cells, and vice versa. Numerical analysis indicated that this was true for ~ 4.4% of the cells originally classified as MLI2 cells, and about 0.8% of the cells originally described as MLI1 cells. Essentially all of these originated from the data set of Kozareva et al. [25]. The reason for this minor discrepancy remains elusive, but may relate to the fact that the original classification of these human cells was done following their integration with murine cells. Be that as it may, we removed these few cells of unresolved classification prior to further analysis (see also below, paragraph “*Nuclear inhibitory interneurons cluster with cortical Golgi*, *MLI2 and PLI neurons”).*

In contrast to the efficient separation of the cell types mentioned above, nuclear neurons of classes i2 and i3 as defined by Kebschull et al. [30] and those from the nuclear preparation of Siletti et al. [31] assigned to their super-cluster “cerebellar inhibitory” overlapped extensively with PLI and MLI2 cells and, to a somewhat lesser degree, also with Golgi cells (Fig. 1A). Importantly, the pattern of overlap between cortical and nuclear cells was not related to the cell preparations integrated (Fig. 1B).

### Inhibitory Neurons Projecting to the Inferior Olive

The co-clustering of the cells originally assigned to cluster 362 by Siletti et al. [31] (referred to below as “cluster 362” cells) with those of cluster i1 of Kebschull et al. [30] (Fig. 1A, B) suggests that these might represent the same cell type. To follow up on this suspicion, we contrasted gene expression in i1 cells and in cluster 362 cells, separately, with all other cells in our sample. This led to the identification of 847 genes in i1 cells expressed in at least 30% of these cells and showing an average log fold change of at least 2 and an adjusted p value of less than 0.05. By the same criteria, we identified 481 genes up-regulated in cluster 362 cells. Finally, when the joint i1 and cluster 362 sets were compared to all other cells in our sample, this approach led to the identification of 639 genes. The 334 genes common to these three comparisons (Supplementary Table 3) define a core set of genes consistently expressed in i1 and cluster 362 cells that distinguish them from cerebellar inhibitory interneurons. For comparison, an analogous search yielded a much smaller set of 48 genes common to the non homologous cluster 362 and i2.1 cell groups (see Supplementary Table 4).

The core gene set common to human i1 and cluster 362 cells includes the homeobox transcription factor DMBX1, which, in the murine cerebellum, is a specific marker for presumptive inferior-olive projecting neurons (see [9] for a detailed discussion) and the transcription factors ZFHX4, EBF3, FOXP2, TOX and GLIS3. It further includes multiple genes associated previously with cell adhesion or axon guidance (e.g.; SLIT1, 2 and 3; SEMA3D, LAMA2, CDH8 and EPHA7 (the latter also strongly expressed in Golgi cells)) and ion transport/exchange (e.g., SLC24A4, CACNA1E, ATP2B4), and the NMDA receptor subunit, GRIN3A.

As the protein encoded by GRIN3A, GluN3A, requires GluN1 (encoded by GRIN1) to be transported to the cell membrane and form functional receptors, we also probed the expression of GRIN1, and also of the GluN2 coding genes GRIn2A-D by i1/cluster 362 cells.

Of the 2130 cells in these groups, 289 (~ 13%) did not express GRIN1; 500 cells (~ 23%) expressed GRIN1 and also any or several of the GRIN2 genes (but not GRIN3A or B); and 1333 cells (~ 63%) expressed GRIN1, at least one gene of GRIN2A-D, and also GRIN3A, or, rarely, GRIN3B. A very few cells were found to express either only GRIN3A or B (4 cells, 0.2%), or neither of the GRIN2 nor GRIN3 genes (again 4 cells). Consistent percentages were obtained when the cells originally described by Kebschull et al. [30] or Siletti et al. [31] were analyzed separately. For comparison, of 1401 murine i1 cells, 36% were Grin1 negative, 27% were positive for Grin1 and any one or several of Grin2a-d, and 33% were positive for Grin1, any one or several of Grin2a-d, and Grin3a and/or Grin3b. As in human inferior olive (IO)-projecting cerebellar nuclear neurons, Grin3b was expressed at low levels and in only a few cells.

### Human Non-Golgi Inhibitory Interneurons from the Granule and Purkinje Cell Layers

Human non-Golgi inhibitory interneurons resident in the granule and Purkinje cell layers (“PLIs”) are so far poorly understood. In fact, whether the cells referred as Lugaro neurons in humans and mice are equivalent may be debated, as may the number of distinct non-Golgi inhibitory interneurons in humans (see [36, 37]; this latter publication also references the few studies describing the histology of large neurons in the granular layer of humans). As documented in the seminal work of Kozareva et al. [25], joint clustering of human and murine cerebellar cortical inhibitory interneurons allows the delineation of a set of cells corresponding to murine PLIs. This provided a starting point to probe the molecular heterogeneity of these cells, and to work towards their classification.

**Fig. 2:**
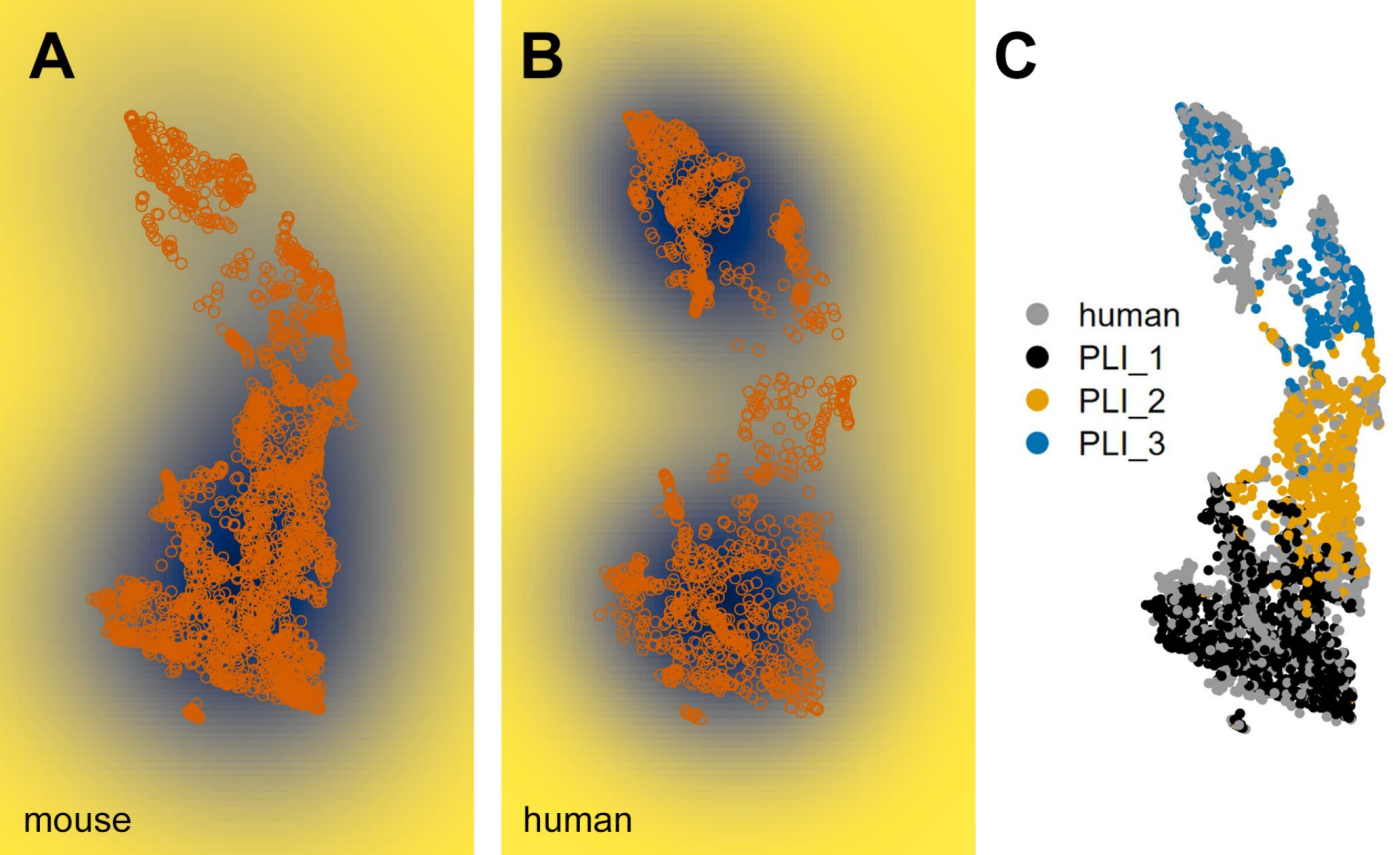
Projection of human PLIs cells into the latent space defined by murine PLIs reveals relative lack of human cells corresponding to murine PLI2 cells. Panels **A** and **B** show cell-density plots for mouse and human PLIs. Panel **C** identifies PLI-subtypes as identified for murine cells. Based on the cell-integration by Kozareva et al. [25], and the subtype classification by Osorno et al. [26]

Strikingly, as already visible in the publication of Kozareva et al. ([25]; their Extended Data Fig. 3), human PLI cells are discretely distributed across the low-dimensional projection space defined by murine PLIs (Fig. 2A-C). Human cells cluster predominantly with murine PLIs of type 1 and 3. This suggests that human PLIs are dominated by cells similar to murine candelabrum (PLI1) and Lugaro cells (PLI3), and comprise only a few cells similar to murine globular cells (PLI2).

**Fig. 3:**
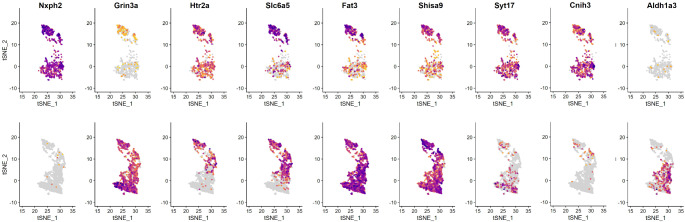
Distribution of cells expressing selected genes by human and murine PLIs. The top row shows expression in human cells, and the bottom row that in murine cells. The tSNE-coordinates of the integrated cells were obtained from Kozareva et al. [25]. Expression strength is color coded, with darker colors indicating stronger expression. Note however that absolute expression levels vary considerably between genes, such that quantitative comparison between different genes is not possible, as the color scale is adjusted to the maximum expression of each gene individually

However, the expression of HTR2A and SLC6A5, which have been used to associate subsets of murine PLIs with candelabrum (PLI1; Htr2a^−^, Slc6a5^−^), globular (PLI2; Htr2a^−^, Slc6a5^+^), and Lugaro cells (PLI3; Htr2a^+^, Slc6a5^+^; see [26]) does not allow a similar subdivision of human PLIs. In contrast to the quite selective expression of Htr2a by murine PLI3 cells, this gene is expressed rather indistinctively in human PLIs (Fig. 3). In fact, whereas in murine samples Htr2a expression allows subdividing a larger Slc6a5-positive population of PLIs [26], in human samples cells positive for HTR2A outnumber those expressing SLC6A5 (634 vs. 428 cells from the data of Kozareva et al. [25]; see Fig. 3). While the data set of Siletti et al. [31] comprises only a few (90) PLIs, of these, too, more (58) are positive for HTR2A than for SLC6A5 (35). Thus, the ratio of HTR2A to SLC6A5 cells for the two data sets is of the same order of magnitude (1.5 and 1.7, respectively).

A search for genes preferentially expressed in either human or murine PLIs identified a number of transcripts present in essentially all (> 95%) human PLIs, but only a small fraction (< 3%) of murine PLIs. These included NXPH2, TTBK2, DNAH14 and VCAN. Supplementary Table 5 gives a more extensive list and comprises also genes broadly expressed in murine PLIs, but in only smaller sets of human PLIs. Among them, we find Grin3a, Cacna1e, Slc24a5, Neurod6 and Nrp1. Of note, Aldh1a3, which was used to differentiate between various subsets of murine PLIs and is expressed preferentially in PLI2 cells of this species [26], is essentially not expressed in human PLIs, though weakly in minor subsets of human MLIs and Golgi cells (not shown). Figure 3 depicts the pattern of cellular expression of some of these genes, together with that of other genes noteworthy for their differential expression in human and murine subsets of PLIs, as defined by the classification of murine PLIs [25, 26]. HTR2A and SLC6A5 were already mentioned above. Moreover, FAT3 and SHISA9 are expressed quite homogeneously in murine PLIs, whereas in human cells, strong expression can only be seen in the subset clustering with murine PLI3 cells. SYT17 [38] is expressed rather homogeneously in human PLIs, but preferentially in murine PLI1 cells. As a last example, CNIH3 may be mentioned, which again is expressed rather homogeneously in human PLIs; in murine cells, it is expressed in a subset of PLI3 cells. In Supplementary Table 6, percentages of cells expressing selected genes or co-expressing selected pairs of genes are listed.

The genes preferentially expressed in either human or mouse PLIs may be subdivided in two groups: one broadly expressed also in other cerebellar inhibitory (inter-) neurons, and a much smaller group showing a more restricted expression in distinct types of cerebellar inhibitory (inter-) neurons other than PLIs (Supplementary Fig. 1). Intriguingly, as documented in Supplementary Fig. 1, the cell type-specificity of these latter genes varied between human and murine cells. Lastly, we note that we could not identify any gene the expression of which would allow a global distinction between human PLIs and nuclear inhibitory interneurons. In mice, Slc34a2, Zfp599 and Trim 30a are candidates to allow such a distinction.

### Nuclear Inhibitory Interneurons Cluster with Cortical Golgi, MLI2 and PLI Neurons

To further characterize the gene-expression-based similarities between human i2 and i3 cells and human cortical MLI2, PLI and Golgi cells suggested by their overlapping clustering, we isolated these from our data set. In this step, we also removed the few cells that had been assigned to these cell types but were clear outliers and clustered mostly with MLI1 cells. This resulted in the elimination of 285 cells, i.e. some 3% of all cells (or some 4.4% originally labeled as MLI2 cells, ~ 0.5% as Golgi cells, ~ 1% as PLIs and 1.1% as nuclear cells from the Siletti et al. [31] data set).

**Fig. 4:**
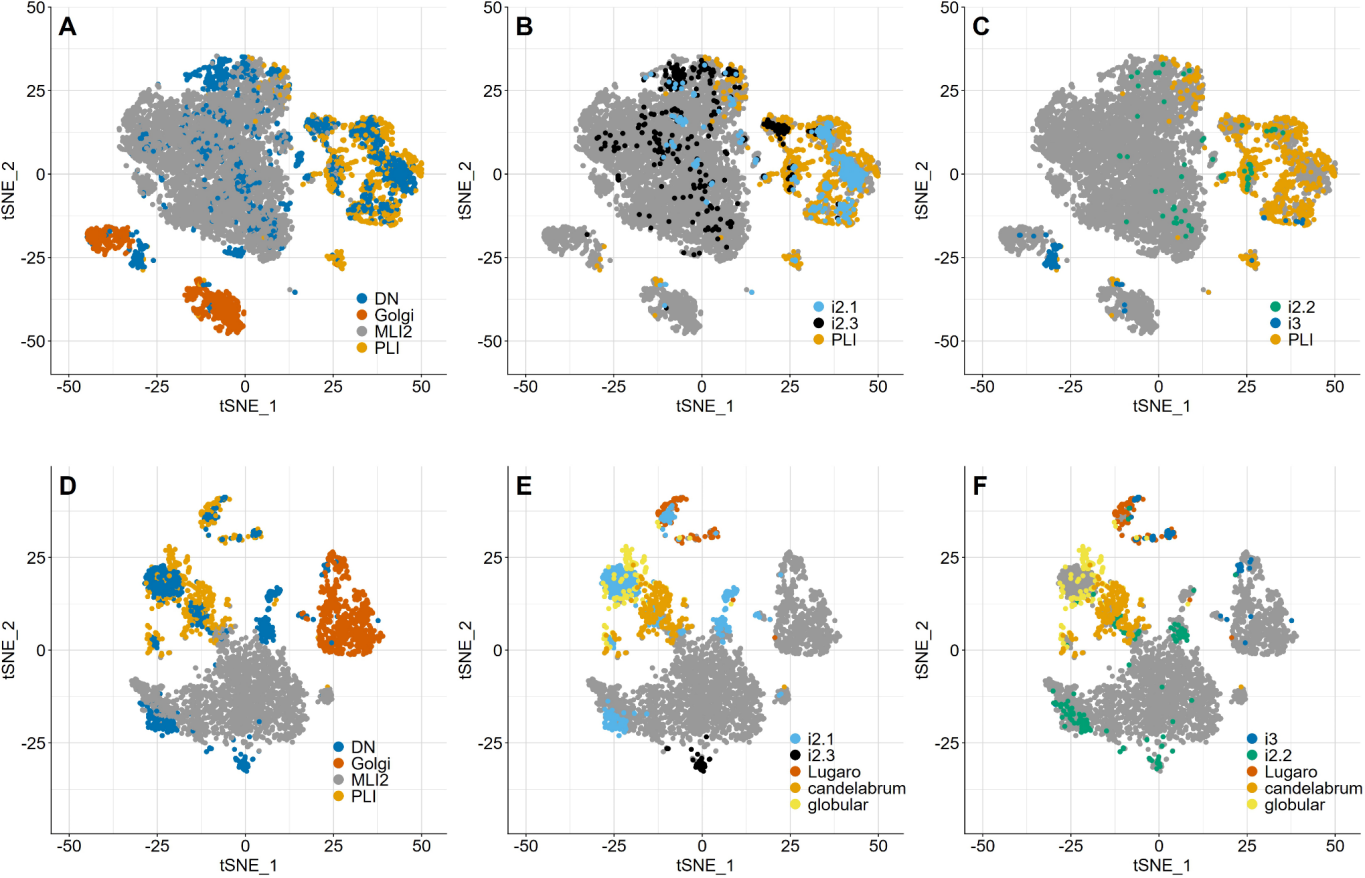
Co-clustering of human and murine nuclear inhibitory interneurons of classes i2 and i3 with cortical PLIs, MLI2 cells, and Gogli cells. Panels A-C show data for human cells, and panels D-F show data for murine cells. In panels **A** and **D**, major cell types/classes are identified for orientation, and panels **B**, **C**, **E** and **F** document the clustering of indentified nuclear cell types. Whereas murine i2.3 cells cluster separately from cortical cells (**E**), human i2.3 cells (**B**) overlap with both MLI2 and a subset of PLI cells. But note also that a set of human i2.3 cells clusters close to, but not with MLI2 cells (at about tSNE coordinates − 8/31), reminiscent of the single i2.3 cluster in mice. i3 cells in both species co-localize with Golgi cells and PLIs, which in mice may be identified as Lugaro cells (**C**, **F**)

The set of human cells thus obtained comprised 5400 MLI2 cells, 931 PLI cells, 907 Golgi cells and 2138 nuclear cells of classes i2 and i3 (including the corresponding cells from the Siletti et al. [31] data set). Their re-integration confirmed the separate clustering of MLI2, PLI, and Golgi cells and the extensive overlap of deep nuclear cells with these three cortical populations (Fig. 4A). Intriguingly, it also revealed that nuclear cells of type i2.1, i2.2, i2.3 and i3 preferentially clustered with distinct sets of cortical inhibitory interneurons. Thus, i2.1 cells were found preferentially within the area defined by PLIs (Fig. 4B). i2.2 and i2.3 (Fig. 4B, C) cells overlapped primarily with MLI2 cells, but also with distinct subsets of PLIs slightly separated from the bulk of PLI cells; the same holds for i2.3, which, in addition, also form a small cluster very close to but not overlapping with MLI2 cells (approximately at coordinates − 8/31 in Fig. 4B). i3 nuclear cells clustered close to Golgi (type 1) cells, where also a minor subset of PLIs was located. A few i3 cells were also found at the margin of the main group of PLIs (Fig. 4C). Of the nuclear inhibitory neurons from the data set of Siletti et al. [31], those originally assigned to supercluster “cerebellar inhibitory” (clusters 298, 299, 300, 302, 307) partitioned with cell types i2.1 - i2.3 of Kebschull et al. [30], whereas those originally assigned to supercluster “Splatter” (clusters 365, 380) clustered with i3 cells (Supplementary Fig. 2C).

To probe whether the transcriptomic likeness of nuclear and cortical cells suggested by their co-clustering might be species specific, we re-analyzed murine data [25, 30] for which we had previously observed a close clustering of cortical and nuclear cells [39]. The present analysis differs from that presented in this latter preliminary publication as it better accounts for sequencing depth; builds on a larger sample of cortical neurons; and uses the Harmony algorithm rather than CCA for integration (for details and references, see Materials and Methods, “*Integration of cortical and nuclear inhibitory interneurons*”). This murine cell set comprised 962 nuclear cells of classes i2 and i3, 437 PLI cells, 1872 MLI2 and 745 Golgi cells.

As observed for human cells, murine i2.1, i2.2 and i3 cells also clustered with MLI2, PLI, and Golgi cells. Similar to the co-clustering pattern seen for human cells, the bulk of murine i2.1 cells clustered with PLI cells. In contrast, those clustering with or close to MLI2 cells were much more concentrated than in humans. Murine i2.2 cells clustered preferentially with MLI2 cells; and murine i3 nuclear cells clustered with both PLIs and close to Golgi cells (Fig. 3D-F).

One eye-catching difference distinguishing human and murine inhibitory interneurons is that in mice, i2.3 cells form a single, distinct cluster close to MLI2 cells (Fig. 4E). In contrast, in the human sample, only a subset of i2.3 cells form a tight cluster close to MLI2 cells (compare Fig. 4A and B; at about coordinates − 8/31), and additional i2.3 cells are found close to PLI cells, and scattered among MLI2 cells. To probe whether this visual impression that human i2.3 cells might be more diverse than their murine homologues, we compared the cross-entropy of MLI2 and i2.3 cells in both species. This measure allows quantitative comparison of low-dimensional representations of global gene expression [76]. Consistent with the visual impression conveyed by Fig. 4B and F, the cross-entropy of murine i2.3 cells was much smaller than that of human i2.3 cells (Supplementary Fig. 4). Importantly, for human cells, the cross-entropy of i2.3 cells was larger than that of MLI2 cells, whereas for murine cells, the cross-entropy of i2.3 cells was smaller than that of MLI2 cells (Holm-adjusted p-values for all comparisons < 0.001). Here, the cross-entropy of MLI2 cells may be taken as a species-specific internal reference. It is also obvious that i2.1 and i2.2 cells are less tightly clustered in humans than in mice. Lastly, the murine data also suggest that distinct subsets of i2.1 cells preferentially clusters with PLIs (tentatively) identified (see [26]) as globular, candelabrum or Lugaro cells (Fig. 4E), and that murine i3 cells cluster preferentially with Lugaro cells (Fig. 4F; note that i2.1 and i3 cells clustering with Lugaro cells do not overlap). A search for genes differentially expressed in the distinct subsets of i2.1 cells co-clustering with various PLIs or MLIs showed that gene expression differences were essentially quantitative and did not reveal any singular specific markers for these subsets.

Finally, the limited numbers of human samples available (see Supplementary Table 1) does not allow assessing whether, and how, cell types vary by sex, age or health status.

## Discussion

The present results provide a gene-expression based comparison of cortical and nuclear inhibitory interneurons in the human cerebellum. They indicate that human non-Golgi inhibitory interneurons resident in the granule and Purkinje cell layers (PLIs) differ from murine PLIs by the expression of key function-predicting genes. Further, they highlight the prominent molecular diversity of human cerebellar nuclear inhibitory interneurons as compared to those of mice, and they reveal molecular resemblances between nuclear inhibitory interneurons and subsets of PLIs, molecular layer resident inhibitory interneurons, and Golgi cells. While the focus of the present analysis is clearly focused on species differences, it also underscores the previously documented molecular similarities of anatomically defined cerebellar inhibitory cell types in humans and mice [25, 30]. To these we may add that in both species, cortical and nuclear interneurons join largely overlapping global patterns of gene expression, as indicated by their extensive overlap when projected into tSNE-space.

### The Nature of the Nuclear Cells Described by Siletti et al

Before discussing our observations in some more detail, a short note on the identity of the cells assigned to supercluster “cerebellar inhibitory” and originating from the cerebellar nuclear dissection reported by Siletti et al. [31] seems warranted. Most of these cells were originally annotated as PLIs, i.e. cells originating from the cerebellar cortex. Siletti et al. cautiously indicate that “distinguishing anatomical borders between dissections was challenging [and dissections…] might contain adjacent tissue”, but available evidence strongly suggests that their cerebellar nuclear dissection was at best minimally, if at all, contaminated by cortical cells. Thus, it comprises only a very few cells that may be tentatively considered as Golgi cells. Also, the original annotation by Siletti et al. [31] implies that their cerebellar nuclear dissection comprises virtually no inhibitory interneurons. This discrepancy between anatomical origin and molecular classification may be traced to the set of genes (TFAP2B, VCAN, GAD2, KLHL1, NXPH1, CDH22, CHRM2, GRIK4, GRM5; see the supplementary Table 3 of Siletti et al. [31]; and https://github.com/linnarsson-lab/auto-annotation-ah/blob/main/Human_adult/Subtype/Neuronal/CB-PLI.md) used for the auto-annotation of these cells. This list was originally compiled for its reliability to distinguish PLIs from other *cortical* cerebellar inhibitory neurons. As an interrogation of the data on cerebellar nuclear inhibitory interneurons of Kebschull et al. [30]; see also https://cellxgene.cziscience.com/d/b8032d54-592d-4b46-b308-fea0ac59013b.cxg/) reveals, these genes are all also expressed in the majority of nuclear inhibitory neurons. Thus, the cells originating from the nuclear dissection of Siletti et al. [31] are bona fide nuclear cells, and not PLIs.

### Cerebellar Nuclear Neurons Projecting to the Inferior Olive (i1 and Cluster 362 Cells) May Comprise Genetically Defined Subgroups

The co-clustering (Fig. 1) of i1 and Splat_362 cells and the common set of genes the up-regulation of which distinguishes them from other nuclear cell types clearly indicates that these two sets belong to the same cell class, which has been likened to nuclear IO-projecting neurons, i.e. the GABAergic cells projecting to the inferior olive [9]. Of the genes preferentially expressed in i1 and cluster 362 cells, FOXP2 appears particularly interesting. Of note, this observation is consistent with immuncytochemical findings in mice showing that cerebellar expression of Foxp2 is not restricted to Purkinje cells, but also found in a subset of deep nuclear neurons [40]. Thus, nuclear i1/cluster 362 cells may yet be another candidate neural substrate for the vocalization deficits associated with Foxp2 mutations or global Foxp2 ablation that cannot be reproduced by Foxp2 deletions in the cerebellar cortex ([41]; note that these authors used the strictly Purkinje cell-specific L7-promotor [42, 43] for ablating Foxp2).

The differential expression of mRNAs coding for various subunits of the NMDA receptor, notably the strong expression of GRIN3A in i1/cluster 362 cells, raises the question whether these cells might be functionally more heterogeneous than hitherto known. Thus, the current data suggest that a smaller fraction (~ 14%) of IO-projecting neurons might not express functional NMDA receptors, as they are negative for the GRIN1; another group (~ 23%) is predicted to express NMDA receptors formed by subunits coded for by GRIN1 and any one or several of the genes GRIN2A-D; and ~ 62% of all IO-projecting neurons express GRIN1, any one or several of the genes GRIN2A-D, and GRIN3A or B. This latter group may thus express NMDA receptors formed by GluN1/GluN3 subunits, which could translate fluctuations of the extracellular glycine concentration into tonic depolarizing currents [44, 45]. They might also form GluN1GluN2/GluN3 receptors, the physiology of which is so far poorly understood [45].

The expression of genes coding for NMDA receptor subunits suggest that IO-projecting cells may be broadly subdivided into three fractions that differ by their sensitivity to glutamatergic feedback from the inferior olive [46, 47] and/or ambient or synaptically released glycine. Lastly, the species specific differences in the expression of Grin2 a and d (Supplementary Fig. 1), if reflected on the protein level, would imply, inter alia, differences in agonist potency, calcium ion permeability and Mg^2+^-blockade (reviewed in [48]), the latter two critical for Hebbian long-term synaptic plasticity (e.g [49]). Unfortunately, whether and how synaptic plasticity is regulated in IO-projecting cerebellar nuclear cells is currently unknown, as these cells remain “poorly explored in terms of intrinsic and synaptic electrophysiology” [50].

### Non-Golgi Inhibitory Interneurons from the Granule Cell and Purkinje Cell Layers of Humans Differ by the Expression of Functionally Interpretable Genes from these Cells in Mice

For humans, as for mice, differential gene expression allowed the ready identification of a set of inhibitory cortical interneurons (referred to as PLIs) clustering well separated from Golgi cells and molecular layer interneurons. Further, these cells could be projected into the latent space defined by murine PLIs using integrative non-negative matrix factorization (iNMF) [25], and consequently are referred to as human PLIs.

Strikingly, Slc6a5, Htr2a and Aldh1a3, previously used to relate molecularly defined subgroups of murine PLIs to physiologically and morphologically characterized candelabrum, globular and Lugaro cells [26] were prominent among the genes differentially expressed between murine and human PLIs. So were genes presumed to fine–tune inhibitory function(s). Thus, ~ 93% of all human PLIs express both GAD1 and GAD2, whereas in mice, ~ 24% of all PLIs express Gad2 only, and ~ 11% express Gad1 only. As the dynamics and regulation of the expression of these two genes differ [51], this might predict differences in the activity dependent GABA-synthesis and thus also impinge on the GABA/glycine ratio in mixed GABA/glycine-ergic PLIs, which account for ~ 50% of all PLIs in humans, and 32% in mice (supplementary Table 6). This ratio has been shown to tune the dynamics and efficiency of inhibitory synapses [52]. We also note that the fraction of glycinergic PLIs in humans is larger than in mice (supplementary Table 6), whereas the converse is true in nuclear interneurons, as pointed out by Kebschull et al. [30]. Any further functional interpretation of these findings will also have to consider translational efficiency, as well as the fact that Gad1 may yield up to 10 splice variants, not all of which code enzymatically active proteins (for a review, see [51]).

PLIs that express HTR2A are not only much more prevalent in humans (~ 74%) than in mice (~ 14.6%); they also differ from the latter by their transmitter phenotype. As may be deduced from the numerical data summarized in supplementary Table 6, ~ 56% of human Htr2a-positive cells are mixed GABA-/glycine-ergic, and ~ 44% are GABAergic only. In mice, ~ 76% of Htr2a positive cells are mixed GABA-/glycine-ergic, and 24% are GABAergic only.

Other genes that show major differences of expression in murine and human PLIs have been related to either human neurological conditions or specific neuronal functions. Thus, dysfunctional TTBK2 causes spinocerebellar ataxia type 11 [53, 54], and mutations of DNAH14 cause neurodevelopmental deficits [55]. VCAN codes for a constituent of perineuronal nets [56], which have been implicated in a range of conditions, from synaptic plasticity to drug dependency to psychosis [57–60]. SHISA9 and CNIH3 code for AMPA-receptor regulatory proteins of the cysteine-knot and cornichon-type, respectively, critical to AMPA-receptor assembly and function [61–63]. As already indicated above, the presence of GRIN3A (or B)-coded subunits in NMDA-receptors profoundly affects their physiology. The plasma membrane Ca^2+^ exporter encoded by ATP2B4 has been found mutated in familial spastic paraplegia [64], and voltage-dependent calcium channels comprising the CACNA1E-encoded subunit α1E have been linked to synaptic plasticity [65], as has been SYT17 [38]. Neurod6 is expressed in the rodent (rat, mouse) cerebellum primarily during development ([66]; see also the Allen Brain Atlas), but maintained in the granule cell layer and at least some cerebellar nuclear cells. Whether it has any role in the diversification of cerebellar inhibitory interneurons, as it has in the spinal cord [67], remains to be seen. FAT3 is part of the cadherin family of cell adhesion molecules [68], and NRP1 regulates axon guidance and inhibitory synapse location in the cerebellum [69]. SLC24A5 is a potassium-dependent sodium/calcium exchanger, the function of which in the CNS is so far not known. Finally, the human-specific expression of NXPH2 is consistent with previous observations that this gene is not expressed in the murine CNS [70].

It should be noted that these species differences in gene expression are not at odds with the efficient iNMF based integration [25], as iNMF, similar to other batch-correction algorithms, emphasizes shared features between data sets [33, 71, 72]. However, a possible limitation when interpreting species differences may arise from the fact that the bulk of PLIs analyzed here (those originally described by Kozareva et al. [25]) originated from only two donors. Further, this sample comprised Golgi cells from only one donor, raising the question whether inadvertent selection may have occurred during tissue preparation. The general agreement of findings obtained with PLIs originating from the Kozareva et al. [25] and Siletti et al. [31] data sets rather argues against this view. Thus, the distribution across the tSNE-defined projection space of PLIs positive for selected, non-ubiquitously expressed genes was the same for cells from either data set as clearly visible in supplementary Fig. 3, despite the limited number (90) of PLIs present in the preparation of Siletti et al. [31]. This rather supports the view that the human samples at hand are representative, and that differences between murine and human PLIs are genuine and not sampling-related.

### Can Cortical Inhibitory Interneurons Inform about Nuclear Cells, and Vice Versa?

The molecular identity of carefully anatomically isolated inhibitory (inter-) neurons of the cerebellar nuclei has yielded deep insight into their evolutionary history and also their potential functional organization [30]. Their distribution about the low-dimensional space defined by tSNE following their integration with cortical inhibitory interneurons as shown here grants yet another perspective on these cells. Thus, the quite distinct distribution of murine and human i2.3 nuclear cells directly supports the previous suggestion [30] that the numerical increase of this cell type in humans went along with their diversification. As cell numbers are known to affect cluster variation [73] when plotted tSNE (or umap) space, the difference in numbers of human and murine i2.3 cells would make it hard to assess their diversity when plotted in isolation in tSNE. However, the “background” provided by their joint integration with cortical cells grants an independent (if not readily quantifiable /scalable) measure of diversity to ascertain this species difference. This conclusion is further supported by comparison of the cross-entropy of these cell types. A similar species difference may also be seen for i2.1 cells, which form several rather tight clusters in mice and humans, but in the latter also comprise a sizable set spread out about the space defined by MLI2 cells. Whether these sub-cluster, which appear to be due primarily to quantitative differences in gene expression, reflect physiological and/or morphological differences of these cells remains to be seen.

The morphology, network integration and physiology of inhibitory interneurons of the cerebellar cortex is better understood than that of cerebellar nuclei, and our understanding of these cells in humans, for obvious reasons, lags behind that of mice. Arguably, one may hope that the transcriptomic similarities and difference between these cells revealed by the present comparison may help to focus further approaches to the many questions still open, in model organisms and, e.g., in human stem-cell derived cerebellar organoids [74, 75].

## Conclusions

The present findings clearly document salient differences of gene expression by presumptive homologous groups of cerebellar inhibitory interneurons and IO-projecting deep nuclear neurons of mice and humans. They also suggest that the classification of non-Golgi inhibitory interneurons resident in the granule cell and Purkinje cell layers (PLIs) reached for the murine cerebellum may need to be modified before being applicable to humans. Importantly, it should be stressed that differences in gene expression cannot predict functional disparity without also considering, say, differential splicing, mRNA-editing, translational efficacy, posttranslational regulation (e.g., phosphorylation) and subcellular protein localisation. Still, the analyses here should contribute to a framework defining promising routes for morphological and functional studies to further address these open questions of cerebellar biology.

## Electronic Supplementary Material

Below is the link to the electronic supplementary material.

Supplementary Figure 1: Expression of genes selected for their differential expression in human and murine PLIs in subsets of cortical and nuclear inhibitory neurons.
Expression in human cells is shown to the left and that in murine cells to the right. Note that for murine cells, PLI subtypes (candelabrum, Lugaro and globular cells) are shown separately. For human cells, expression in cells originating from the sample of Kozareva et al. [25] and those from the sample of Siletti et al. [31] are shown separately, allowing a comparison of these two samples. As nuclear cells corresponding to cell types i2 and i3 of Kebschull et al. [30] are not further specified in the sample of Siletti et al. [31], their combined expression values are shown as "dn_Si". Also, Golgi cells in the sample of Siletti et al. [31] were not further specified as type 1 or 2.

Supplementary Figure 2: Integration of cerebellar nuclear inhibitory interneurons described by Kebschull et al. and Siletti et al.
Integration of i2 and i3 cells from Kebschull et al. [30] and nuclear cells of clusters 298, 299, 300, 302, 307, 365 and 380 from Siletti et al. [31] revealed their extensive overlap. In panel A, cells originating from Siletti et al. are shown in light gray, and (sub) classes i2.1-i3 are color coded. Panel B gives an overview with cells colored by source. As visible in panel C, cells of the supercluster "Splatter" (clusters 362 and 380) of Siletti et al. [31] cluster with i3 cells as defined by Kebschull et al. [30], whereas cells of supercluster "cerebellar inhibitory" (clusters 298, 299, 300, 302and 307) overlap with i2 cells.

Supplementary Figure 3: Distribution of PLIs from the data sets of Kozareva et al. and Siletti et al. expressing selected genes across the tSNE defined plane.
PLIs were subset from the data shown in Figure 4 and are plotted separately by their origin (upper row, data form Kozareva et al. [25], lower row, data from Siletti et al, [31]) . Note that cells positive for a given gene may be found at comparable positions in the tSNE plane, irrespective of their origin from the two datasets integrated. Expression strength is color coded, with darker colors indicating stronger expression. Note however that absolute expression levels vary considerably between genes, such that quantitative comparison between different genes is not possible, as the color scale is adjusted to the maximum expression of each gene individually.

Supplementary Figure 4: Cumulative density distributions of the cross-entropy of i2.3 and MLI2 cells from humans and mice.
Cross-entropy is a measure sensitive to cluster size (number of cells) and cluster structure (see reference [76] for details). Note that cross-entropy of murine i2.3 cells (i2.3_m) is shifted towards lower values when compared to that of mouse MLI2 cells (MLI2_m), and also when compared to that of human i2.3 cells (i2.3). In contrast, cross entropy of human i2.3 cells is shifted towards larger values when compared to human MLI2 cells (MLI2). These differences are all significant (Holmadjusted p values all < 0.001). If the cross-entropy would reflect sample sizes alone the curves shown should be ordered as i2.3_m < i2.3 < MLI2_m < MLI2 (for cell numbers, see supplementary tables 1 and 2). Thus these data imply structural differences between human and murine i2.3 clusters, reflecting the distinct diversity of human compared to murine i2.3 cells.

Supplementary Table 1: Classification of the human cells analyzed here based on their annotations in the original publications. MLI1 and 2 indicates molecular layer inhibitory interneurons of type 1 and 2, respectively. PLI, Purkinje cell layer inhibitory interneuron. For MLIs, PLIs, and Golgi cells, upper case (first) letters are used to identify data originally published by Kozareva et al. [25], and lower case letters to indicate those of Siletti et al. [31]. The latter are also marked by the suffix "_Si". Nuclear cells originally described by Kebschull et al. [30] are indicated using his classification (i1 – i3), and those originating from the cerebellar nuclear dissection of Siletti et al. [31] are designed as "cluster 362" and "dn_Si". Note that the latter cells were originally described as PLIs, due to a questionable automatic annotation (see main text). Data on donors, as far as known (sex, age, cause of death) are given once per cell sample.
Supplementary Table 2: Classification of the murine cells analyzed here based on their annotations in the original publications. MLI1 and 2 indicates molecular layer inhibitory interneurons of type 1 and 2, respectively. PLI, Purkinje cell layer inhibitory interneuron. Nuclear cells originally described by Kebschull et al. [30] are indicated using his classification (i1 – i3). Donor characteristics (sex, age) are given once per cell sample.
Supplementary Table 3: Core genes distinguishing i1 and cluster 362 cells from inhibitory interneurons of the cerebellar cortex and nuclei. These genes were identified by intersecting genes consistently found upregulated when i1, cells, cluster 362 cells, and the joint set of these cells were contrasted with the cerebellar inhibitory interneurons. Genes included in this list had to be expressed in at least 30% of i1 and/or cluster 362 cells in these comparisons, had to show an average log-fold change of at least 2, and an adjusted p value of less than 0.05. Log-fold changes (av_log2FC) and adjusted p-values (p_val_adj) shown in the table are those from the comparison of the joint sets of i1 and cluster 362 cells with all other cells. "pct.1" indicates the fraction of i1/cluster 362 cells expressing the gene listed, and "diff" indicated the difference of his frequency with the frequency observed in all other cells ("pct.2").
Supplementary Table 4: Core genes distinguishing cluster 362 and i2.1 cells from the other inhibitory interneurons of the cerebellar cortex and nuclei These genes were identified by intersecting genes consistently found upregulated in i2.1 and cluster 362 cells as described in the legend to supplementary table 3 for i1 and cluster 362 cells and may serve as a control for the close similarity of the latter cells cells documented by the data in supplementary table 3.
Supplementary Table 5: Genes preferentially expressed in either human or murine PLIs. Listed are the top 100 genes expressed preferentially in human, and also the top 100 genes preferentially expressed in murine PLIs. Note that the list also includes genes broadly expressed in murine PLIs, but only smaller sets of human PLIs. "frac_human" and "frac_mouse" indicate the fraction of cells expressing a gene in the human and mouse PLI samples analyzed, and "difference" the difference between these two values. Positive values for the log2-fold change (avg_Log2FC) indicate stronger expression in human cells, and negative values stronger expression in murine cells.
Supplementary Table 6: Percentages of human and murine PLIs expressing or co-expressing selected genes Row names "gene1" and "gene2" refer to the first and second gene in the column-titles, respectively.

## Data Availability

No datasets were generated or analysed during the current study.
