# Supplementary figures and images for: A Gene-Expression Based Comparison of Murine and Human Inhibitory Interneurons in the Cerebellar Cortex and Nuclei
Source: Cerebellum. 2025 Feb 28;24(2):55. doi: 10.1007/s12311-025-01809-y (PMC11870911; doi:10.1007/s12311-025-01809-y)

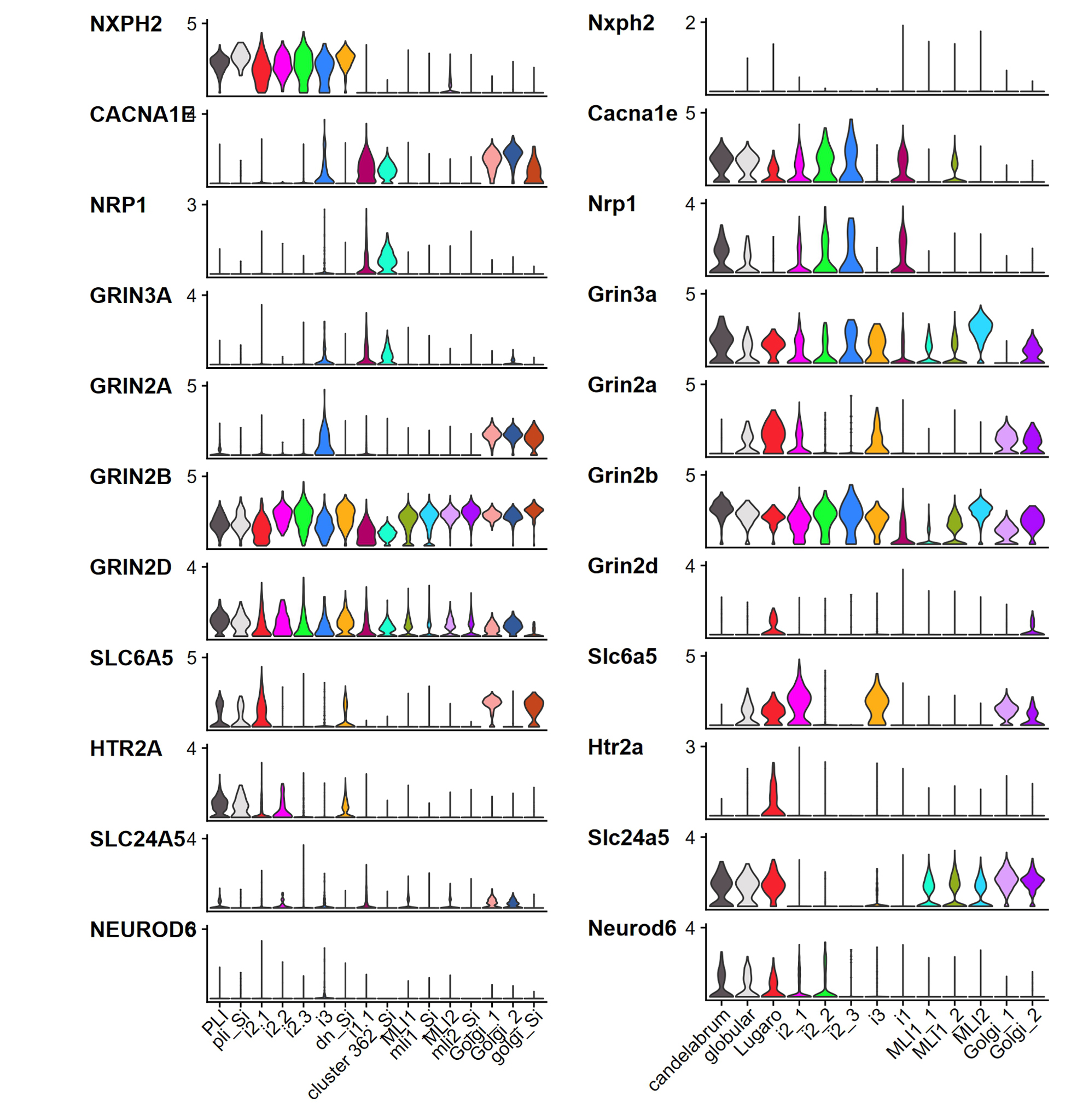

Supplement: Supplementary file 1 — Supplementary Figure 1: Expression of genes selected for their differential expression in human and murine PLIs in subsets of cortical and nuclear inhibitory neurons. Expression in human cells is shown to the left and that in murine cells to the right. Note that for murine cells, PLI subtypes (candelabrum, Lugaro and globular cells) are shown separately. For human cells, expression in cells originating from the sample of Kozareva et al. [25] and those from the sample of Siletti et al. [31] are shown separately, allowing a comparison of these two samples. As nuclear cells corresponding to cell types i2 and i3 of Kebschull et al. [30] are not further specified in the sample of Siletti et al. [31], their combined expression values are shown as "dn_Si". Also, Golgi cells in the sample of Siletti et al. [31] were not further specified as type 1 or 2. [file 12311_2025_1809_MOESM1_ESM.jpg]

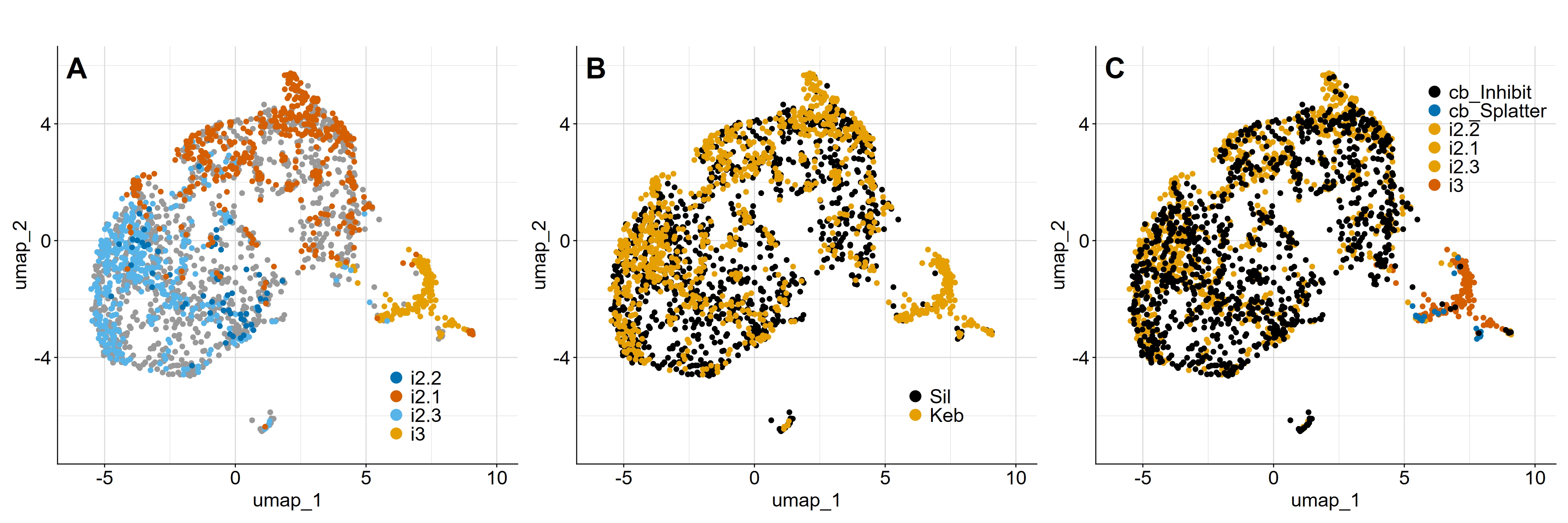

Supplement: Supplementary file 2 — Supplementary Figure 2: Integration of cerebellar nuclear inhibitory interneurons described by Kebschull et al. and Siletti et al. Integration of i2 and i3 cells from Kebschull et al. [30] and nuclear cells of clusters 298, 299, 300, 302, 307, 365 and 380 from Siletti et al. [31] revealed their extensive overlap. In panel A, cells originating from Siletti et al. are shown in light gray, and (sub) classes i2.1-i3 are color coded. Panel B gives an overview with cells colored by source. As visible in panel C, cells of the supercluster "Splatter" (clusters 362 and 380) of Siletti et al. [31] cluster with i3 cells as defined by Kebschull et al. [30], whereas cells of supercluster "cerebellar inhibitory" (clusters 298, 299, 300, 302and 307) overlap with i2 cells. [file 12311_2025_1809_MOESM2_ESM.jpeg]

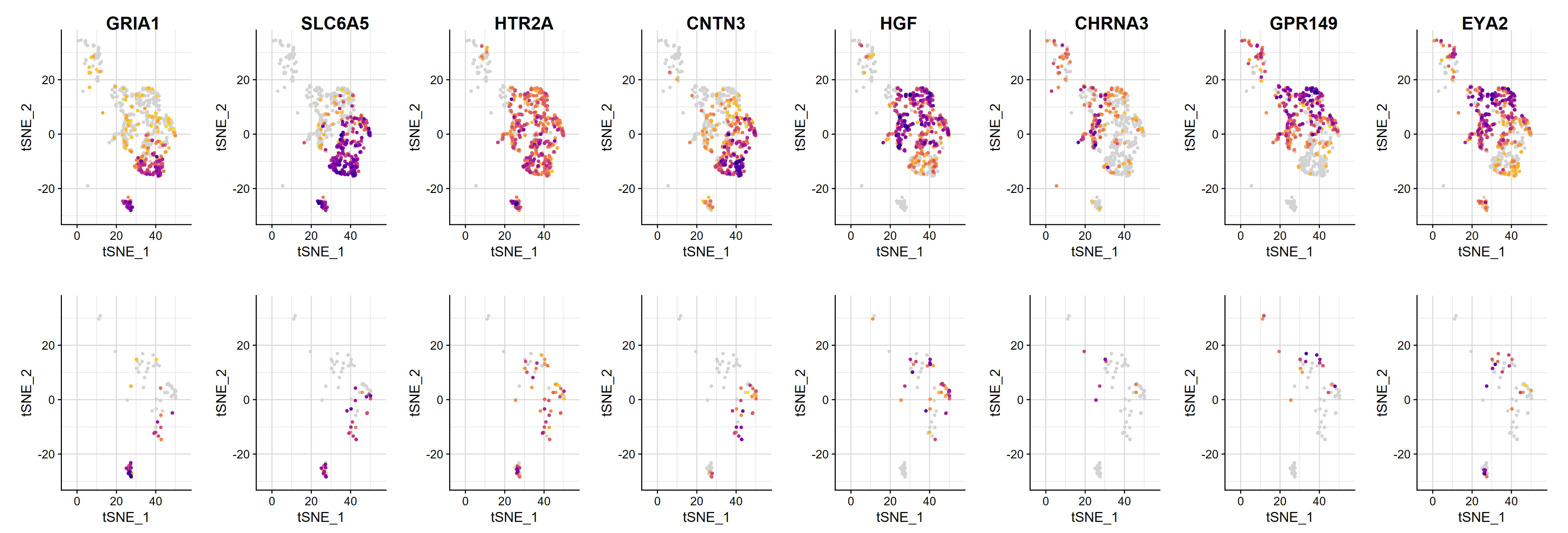

Supplement: Supplementary file 3 — Supplementary Figure 3: Distribution of PLIs from the data sets of Kozareva et al. and Siletti et al. expressing selected genes across the tSNE defined plane. PLIs were subset from the data shown in Figure 4 and are plotted separately by their origin (upper row, data form Kozareva et al. [25], lower row, data from Siletti et al, [31]) . Note that cells positive for a given gene may be found at comparable positions in the tSNE plane, irrespective of their origin from the two datasets integrated. Expression strength is color coded, with darker colors indicating stronger expression. Note however that absolute expression levels vary considerably between genes, such that quantitative comparison between different genes is not possible, as the color scale is adjusted to the maximum expression of each gene individually. [file 12311_2025_1809_MOESM3_ESM.jpg]

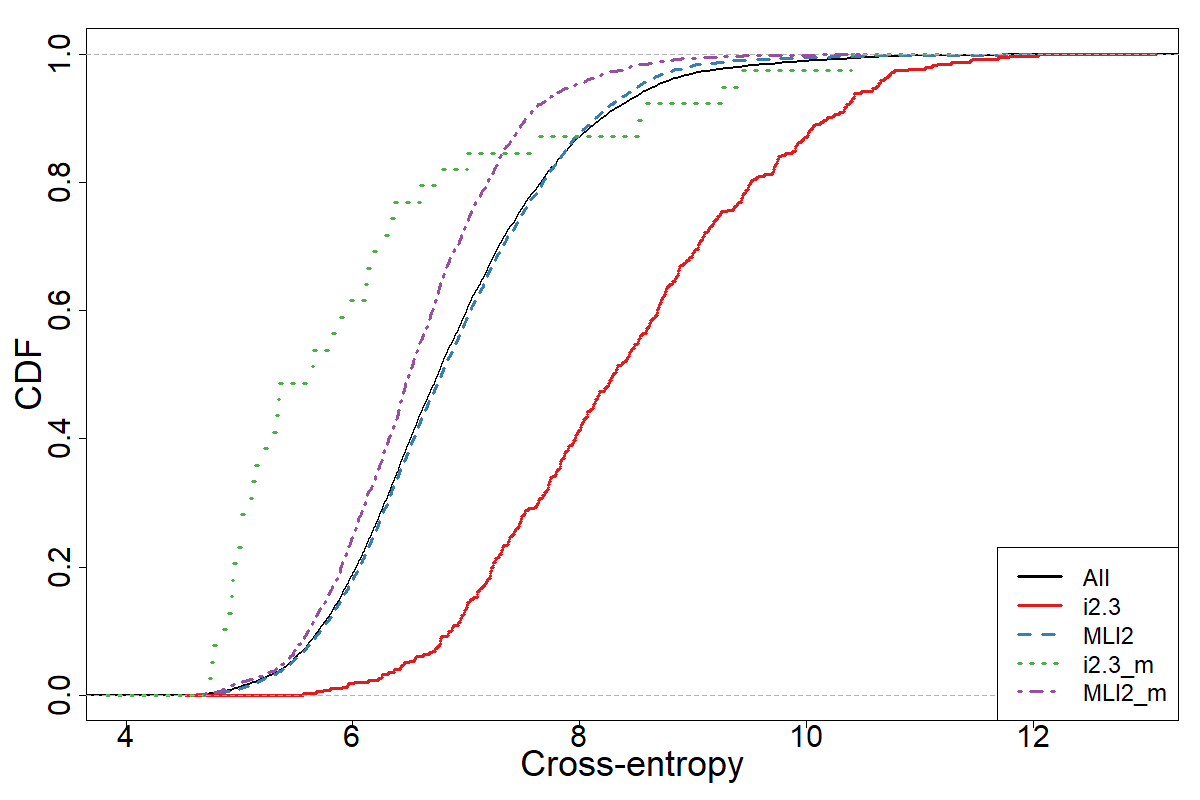

Supplement: Supplementary file 4 — Supplementary Figure 4: Cumulative density distributions of the cross-entropy of i2.3 and MLI2 cells from humans and mice. Cross-entropy is a measure sensitive to cluster size (number of cells) and cluster structure (see reference [76] for details). Note that cross-entropy of murine i2.3 cells (i2.3_m) is shifted towards lower values when compared to that of mouse MLI2 cells (MLI2_m), and also when compared to that of human i2.3 cells (i2.3). In contrast, cross entropy of human i2.3 cells is shifted towards larger values when compared to human MLI2 cells (MLI2). These differences are all significant (Holmadjusted p values all < 0.001). If the cross-entropy would reflect sample sizes alone the curves shown should be ordered as i2.3_m < i2.3 < MLI2_m < MLI2 (for cell numbers, see supplementary tables 1 and 2). Thus these data imply structural differences between human and murine i2.3 clusters, reflecting the distinct diversity of human compared to murine i2.3 cells. [file 12311_2025_1809_MOESM4_ESM.png]
